# A Practical Treatise on Diseases of the Skin

**Published:** 1888-04

**Authors:** 


**A Practical Treatise on Diseases of the Skin.** By JOHN V. SHOEMAKER, Professor of Skin and Venereal Diseases in the Medico-Chirurgical College and Hospital of Philadelphia, etc., etc. With colored plates and other illustrations. New York: D. Appleton & Co., 1888.

The author of this book is one of the well-known dermatologists of this country, and has had exceptional opportunities for observation and study in his special department. These advantages have enabled him to prepare a book admirable in its clearness of description, conciseness and thoroughness. Dr. Shoemaker has introduced into the treatment of skin diseases many of the oleates, and, consequently, we find that these preparations receive much attention and are highly lauded. That they are important additions to our resources, is generally admitted, but we think the author often overestimates their value. Under the treatment of favus of the scalp, for instance, we find him depending upon the application of oleate of mercury or copper, and referring to the treatment by epilation as "barbarous." Other means of treatment are, however, not ignored, and as a whole the work deserves high commendation.

